# Supplementary material for: Psychosocial Health of K-12 Students Engaged in Emergency Remote Education and In-Person Schooling: A Cross-Sectional Study
Source: Int J Environ Res Public Health. 2021 Aug 13;18(16):8564. doi: 10.3390/ijerph18168564 (PMC8394738; doi:10.3390/ijerph18168564)
Supplement: Supplementary file 1 [file ijerph-18-08564-s001.zip › ijerph-1297007-supplementary.pdf]

SECTION A

Date: _____________

Date: _____________
Child Name____
Address____
____

Contact information for follow-up:
Name________ [NAME Variable used in the following questions]
phone ____________
e-mail _____________

Child Age (years) _______

Date of Birth (MM/DD/YYYY)_______

Child's biological sex Male ____ Female ____

Is [NAME] Hispanic or Latino? (Check only one)

- ❑ Yes, my student is Hispanic/Latino -- a person of Cuban, Mexican, Puerto Rican, South or Central American, or other Hispanic culture or origin, regardless of race
- ❑ No, my student is not Hispanic/Latino

Please check ALL racial descriptors that apply to [NAME].

- ❑ American Indian or Alaska Native -- a person having origins in any of the original peoples of North and South America (including Central America) and who maintains tribal affiliation or community attachment
- ❑ Asian -- a person having origins in any of the original peoples of the Far East, Southeast Asia, or the Indian subcontinent, e.g., Cambodia, China, India, Japan, Korea, Malaysia, Pakistan, the Philippine Islands, Thailand, and Vietnam
- ❑ Black or African American -- a person having origins in any of the black racial groups of Africa
- ❑ Native Hawaiian or Other Pacific Islander -- a person having origins in any of the original peoples of Hawaii, Guam, Samoa, or other Pacific Islands
- ❑ White -- a person having origins in any of the original peoples of Europe, the Middle East, or North Africa

How many years of school did you complete? _____ years

What type of health insurance does your child have?

Private _____
Medicare _____
Medicaid _____
None/Self-pay _____

Does your child have any of the following chronic illnesses?

Seasonal allergies ______
Asthma _____
Diabetes _____
Obesity _____
Seizure disorder (epilepsy) ________
Sicke cell disease ________
Immunosuppression ______ If immunosuppressed, why? ______________
Prematurity (born prior to 37 weeks gestation) ___________
Other _____ Specify: ____

[If child is under 14]
Does your child live with an adult who smokes cigarettes? Yes____ No_____

If yes, how many packs does this person smoke per day? _______

[If child is over 14]
To your knowledge, does your child smoke cigarettes? Yes_____ No_____

To your knowledge, does your child use electronic cigarettes/vaping products? Yes _____ No ______

[Back to all]
What medications does your child take regularly (both prescription and "over the counter" non-prescription)?

Medication	Dose (mg, if known)	Number of times your child takes it each day

Flu history (influenza)
Has your child ever received a flu vaccine? (y/n)
Did your child receive the flu vaccine last year (Sept 2019-May2020)? (y/n)
If yes, injection or nasal?
Was your child diagnosed with the flu last year (Sept 2019-May2020)? (y/n)
If yes, when? (mm/dd/yy)
If yes, were they hospitalized for the flu ? (y/n)

Work
Is any adult in your child's home currently employed? Yes ____ No _____
[IF YES]
For the adult with the steadiest employment, is s/he part time or full time?
Full time____ Part time ______

Does anyone in your child's home work or volunteer in a hospital or other medical setting?
Yes ____ No _____

[IF YES], where do they work/volunteer (enter all that apply)? ____________________________

[IF NO], where does the adult work or volunteer (enter all that apply)? ___________________________

Has a primary wage earner within your child's household lost their job or regular income source due to COVID-19? Yes ____ No _____

Home
Does your child live in an apartment complex or other multi-unit type of dwelling? Yes ____ No ____
[IF YES], provide details: ___________________________

SECTION B

Knowledge questions

In yellow the ones I think we should keep, in green the new ones

For the following statements, please indicate if True or False.

K1. The main clinical symptoms of COVID-19 are fever, fatigue, dry cough, and muscle aches.
K2. Unlike the common cold, stuffy nose, runny nose, and sneezing are less common in persons infected with the COVID-19 virus.
K3. There currently is no effective cure for COVID-19, but early symptomatic and supportive treatment can help most patients recover from the infection.
K4. Antibiotics can be used to treat COVID-19
K5. Not all persons with COVID-19 will develop to severe cases.
K6. People of all racial and ethnic groups can become infected with the COVID-19 virus.
K7. Most people who are infected with the COVID-19 virus recover from it
K8. Handwashing can help reduce transmission of the COVID-19 virus.
K9. Persons with COVID-19 cannot pass the virus to others if they do not have symptoms.
K10.The COVID-19 virus spreads via respiratory droplets from infected individuals.
K11. People can wear general medical masks to prevent infection by the COVID-19 virus.
K12. It is not necessary for children and young adults to take measures to prevent infection by the COVID-19 virus.
K13. Isolation and treatment of people who are infected with the COVID-19 virus are effective ways to reduce the spread of the virus.
K14. People who have contact with someone infected with the COVID-19 virus should be immediately isolated in a proper place for 14 days.
K15. People with a strong immune system will not get infected with COVID-19

K16. To prevent the infection by COVID-19, individuals should avoid going to crowded places such as restaurants, bars, concerts, etc.

Attitudes about COVID-19

For the following statements, please indicate on a scale of 1-5, with 1 being strongly disagree and 5 being strongly agree

1 – Strongly disagree

2 – Disagree
3 – Neither agree nor disagree
4 – Agree
5 – Strongly agree

A1. I am worried about getting infected with the COVID-19 virus.

A2. I feel confident I can prevent myself and my family from becoming infected with the COVID-19 virus.

A3. I know what actions to take to prevent myself and my family from becoming infected with the COVID-19 virus.

A4. I support CDC imposed guidelines for those who are infected with the COVID-19 virus.

A5. I support/would support city and state-imposed regulations to protect the public (e.g. business closures, mask wearing, park closures, beach closures, etc.)

A6. I support postponing or canceling mass gatherings such as concerts, festivals, and sporting events.

A7. I support closure of K-12 schools if any student, staff member, or teacher is found to have COVID-19.

A8. If I were exposed to and could possibly be infected with the COVID-19 virus, I would be willing to quarantine myself at home for 2 weeks until I was sure I was not infected, in order to prevent others from getting COVID-19 from me.

A9. I support K-12 schools re-opening in person instruction this fall.

A10. I am comfortable going out in public as long as I am in open spaces.

A11. I am comfortable going into essential public spaces as necessary, such as grocery stores, pharmacies, etc.

A12. I am comfortable going into public retail spaces, such as department stores, restaurants, etc.

A13. I think that the US government is handling the COVID-19 health crisis well.

A 14. I support mask mandates in closed-in areas where physical distancing (6 ft) is not feasible (stores, offices, schools, etc.)

Practices about COVID-19

P1. In recent days, I am washing my hands with soap and water more often than normal?

P2. In recent days, I am using more disinfectants, such as hand sanitizers and cloth wipes?

P3. In recent days, I am avoiding shaking hands or other physical contact with others outside my home?

P4. In recent days, I have adhered to social distancing guidelines, such as avoiding meetings of more than 10 people and keeping a distance of 6 feet apart?

P5. In recent days, I have bought larger amounts of staple foods (flour, sugar, pasta, rice, canned food) than normal?

P6. I am avoiding going into any crowded place.

P7. I wear a mask when leaving home and entering an indoor area with other people.

P8. In the past 7 days I have stayed at home or worked from home rather than going into work.

Income

Q3) Total Family Annual Income (use 2019 estimate) $ _________________

PARENTS STRESS and COPING MECHANISMS

In the past three weeks have you had the following feelings or thoughts? *(Never, Occasionally, Half the Time, Often, Always; 1-5)*

1. I worry about many different things
2. I am irritable and have a short temper
3. I have unexplained physical ailments (headaches, digestive problems, dizziness)
4. I am having a hard time: sleeping, eating, interacting with others positively

Problem focused coping
Please indicate whether you agree or disagree with the following statements as they relate to COVID-19 Likert Scale, 1-5, options in green Strongly Disagree, Disagree, Not Sure, Agree, Strongly Agree]

1. I listen to scientific experts and follow their advice.
2. I think carefully about what to do and stick to it.
3. I try not to do anything impulsive.
4. I focus on what to do next.
5. I focus on what I will do next.
6. I talk to others to learn more about the situation.
7. I know what to do and try to do everything with twice the effort.
8. I change things in my life to be able to cope better with it all.
9. I have repeatedly thought about it and try to understand it.
10. I have been thinking about what I usually do with other viral infections.
11. I talk to someone who knows about it.
12. I am doing something completely new that I would never have done in other circumstances.
13. I ask for advice from highly respected people and adhere to it.
14. I have seen something like this before.

Emotional coping
Please indicate whether you agree or disagree with the following statements as they relate to COVID-19 Likert Scale, 1-5, options in green Strongly Disagree, Disagree, Not Sure, Agree, Strongly Agree]

1. It will emerge over time; there is nothing more to do but wait.
2. I turn to my work or other activities to distract myself.
3. I imagine how the whole thing could end.
4. I imagine things that improve my mood.
5. I submit to my fate; sometimes you are just unlucky.
6. I tell myself things that make it easier for me.
7. I do things that are probably of no use, but I feel like at least I am doing something.
8. I wish I could change my worries and feelings.
9. I hope for a miracle.
10. I try to make myself feel better by eating, drinking, smoking or taking medication.
11. I take refuge in daydreams and imagine times when it was better than today.
12. I try to leave the whole thing behind and want to rest or go on vacation.
13. I refuse to believe what is happening.

Vaccine Hesitancy Questions

Primary Outcome Question

V1. Do you plan on having your child(ren) vaccinated against COVID-19 (coronavirus/SARS-CoV-2) when a vaccine becomes available?

- Yes
- Unsure/Haven't Decided Yet
- No

Dimensions of Vaccine Hesitancy/Acceptance Questions

Perceived Vaccine Safety

V2. How concerned are you that the coronavirus vaccine may not be safe for your child(ren)?

- Very Concerned
- Somewhat Concerned
- Not Sure
- Not Too Concerned
- Not at All Concerned

V3. How concerned are you that your child(ren) may have a serious side effect from the coronavirus vaccine?

- Very Concerned
- Somewhat Concerned
- Not Sure
- Not Too Concerned
- Not at All Concerned

Trust

V4. The information I receive about the coronavirus vaccine from the news/media is reliable and trustworthy.

- Strongly Disagree
- Disagree
- Not Sure
- Agree
- Strongly Agree

V5. I am able to openly discuss my concerns about the coronavirus vaccine with my child(ren)'s doctor.

- Strongly Disagree
- Disagree
- Not Sure
- Agree
- Strongly Agree

Perceived Effectiveness/Necessity

V6. The coronavirus vaccine will be effective at preventing COVID-19.

- Strongly Disagree
- Disagree
- Not Sure
- Agree
- Strongly Agree

V7. Having my child(ren) vaccinated against the coronavirus is important for the health of others in my community.

- Strongly Disagree
- Disagree
- Not Sure
- Agree

- Strongly Agree

CDC HRQOL

1. Would you say that in general your health is:
   - ❑ Excellent
   - ❑ Very Good
   - ❑ Good
   - ❑ Fair
   - ❑ Poor

2. Now thinking about your physical health, which includes physical illness and injury, for how many days during the past 30 days was your physical health not good?
   a) ___________ (Number typed in)
   b) None (Skip to next)

3. Now thinking about your mental health, which includes stress, depression, and problems with emotions, for how many days during the past 30 days was your mental health not good?
   a) ___________(Number typed in)
   b) None (Skip to next)

4. During the past 30 days, for about how many days did poor physical or mental health keep you from doing your usual activities, such as self-care, work, or recreation?
   a) ___________(Number typed in)
   b) None (Skip to next)

Optimism

Response scale is the same for questions 1-10 below

- I agree a lot
- I agree a little
- I neither agree nor disagree
- I disagree a little
- I disagree a lot

1. In uncertain times, I usually expect the best.

2. It's easy for me to relax.

3. If something can go wrong for me, it will.

4. I'm always optimistic about my future.

5. I enjoy my friends a lot.

6. It's important for me to keep busy.

7. I hardly ever expect things to go my way.

8. I don't get upset too easily.

9. I rarely count on good things happening to me.

10. Overall, I expect more good things to happen to me than bad.

Other Questions

H1. How easy or difficult would you say it is to:

7-point Likert scale [Very difficult - Very easy]

1. Find the information you need related to COVID-19?
2. Understand information about what to do if you think you have COVID-19?
3. Judge if the information about COVID-19 in the media is reliable
4. Understand restrictions and recommendations of authorities regarding COVID-19
5. Follow the recommendations on how to protect yourself from COVID-19
6. Understand recommendations about when to stay at home from work/school, and when not to?
7. Follow recommendations about when to stay at home from work/school, and when not to?
8. Understand recommendations about when to engage in social activities, and when not to?
9. Follow recommendations about when to engage in social activities, and when not to?
10. Obtain a COVID-19 Test

H2. COVID-19 to me feels ...

a) Close to me - Far away from me [7-point Likert scale]
b) Spreading slowly - Spreading fast [7-point Likert scale]
c) Something I think about all the time - Something I almost never think about [7-point Likert scale]
d) Scared - Not scared [7-point Likert scale]
e) Media hyped -Not media hyped [7-point Likert scale]
f) Something that makes me feel helpless - Something I am able to combat with my own action [7-point Likert scale]
g) Stressful - Not stressful [7-point Likert scale]

H3. How much do you trust information about COVID-19 from the following sources?

7-Point Likert Scale [Very little trust - A great deal of trust]

a) Television
b) Newspapers
c) Health workers
d) Social media
e) Radio
f) UF Health
g) Florida Department of Health
h) Centers for Disease Control and Prevention

i) Celebrities and social media influencers
j) World Health Organization (WHO)
k) COVID-19 Hotlines
l) US Government's National COVID-19 information website

H4. How often do you use the following sources for information about COVID-19?

7-Point Likert Scale [Never -Very often]

a) Television
b) Newspapers
c) Health workers
d) Social media
e) Radio stations
f) UF Health
g) Florida Department of Health
h) Centers for Disease Control and Prevention (CDC)
i) Celebrities and social media influencers
j) World Health Organization (WHO)
k) COVID-19 Hotlines
l) US Government's National COVID-19 information website

H5. How much confidence do you have that the following can handle the COVID-19 challenge well?

7-Point Likert Scale with NA option
[Very low confidence-Very high confidence] // Not applicable

a) Your family doctors
b) Your employer
c) Local hospitals
d) UF Health
e) Florida Department of Health
f) Centers for Disease Control and Prevention
g) Public schools
h) Public transportation companies
i) Police
j) Church/places of worship

H6. Answer the questions below whether you agree or disagree.

7-Point Likert Scale [Strongly disagree - Strongly agree]

a) I have a hard time making it through stressful events
b) It does not take me long to recover from a stressful event
c) It is hard for me to snap back when something bad happens

H7. Please now give your opinion on the following statements:

7-Point Likert Scale [Completely disagree -Completely agree]

a) If a COVID-19 vaccine becomes available and is recommended for me, I would get it.
b) In the event of an outbreak it is appropriate to avoid certain people on the basis of their race/ethnicity and associated risk
c) I think that the restrictions currently being implemented are greatly exaggerated

d) The government should be allowed to force people into self-isolation if they have been in contact with someone who was infected with coronavirus.
e) More tests for coronavirus infection should be carried out in the population
f) I am worried that the pandemic will have economic consequences for me in the future

H8. Please indicate if you have:
Possible Answers [Yes/ No/ Not applicable]

a) Avoided people based on their ethnicity who I thought might infect me
b) Exercised less than I did before the pandemic
c) Drank more alcohol than I did before the pandemic
d) Ate more unhealthy food than I did before the pandemic
e) Smoked more than I did before the pandemic
f) Postponed vaccination for myself or my child
g) Avoided going to the doctor for a non-COVID-19-related problem
h) Bought drugs that I heard are good for treating COVID-19

Food Security

F1. “We have relied on only a few kinds of low-cost food to feed our child/children because we were running out of money to buy food.” Was that often, sometimes, or never true for your household in the last 6 months?
- ❑ Often true
- ❑ Sometimes true
- ❑ Never true
- ❑ Don’t know

F2. “We couldn’t feed our child/children a balanced meal, because we couldn’t afford that.” Was that often, sometimes, or never true for your household in the last 6 months?
- ❑ Often true
- ❑ Sometimes true
- ❑ Never true
- ❑ Don’t know

F3. "Children in my house were not eating enough because we just couldn't afford enough food." Was that often, sometimes, or never true for (you/your household) in the last 6 months?
- ❑ Often true
- ❑ Sometimes true
- ❑ Never true
- ❑ Don’t know

Screener for Stage 2 Child Referenced Questions: If affirmative response (i.e., "often true" or "sometimes true") to one or more of the 3 questions above, then continue to Child Stage 2; otherwise skip to End.

F4. In the last 6 months, since April 2020, did you ever cut the size of meals for a child in your house because there wasn't enough money for food?

- ❑ Yes
- ❑ No
- ❑ Don’t know

F5. In the last 6 months, did a child in your house ever skip meals because there was not enough money for food?

- ❑ Yes
- ❑ No
- ❑ Don’t know

F5a. [IF YES ABOVE ASK] How often did this happen—almost every month, some months but not every month, or in only 1 or 2 months?

- ❑ Almost every month
- ❑ Some months but not every month
- ❑ Only 1 or 2 months
- ❑ Don’t know

F6. In the last 6 months, were any of your children ever hungry but you just couldn't afford more food?

- ❑ Yes
- ❑ No
- ❑ Don’t know

F7. In the last 6 months, did any of your children ever not eat for a whole day because there wasn't enough money for food?

- ❑ Yes
- ❑ No
- ❑ Don’t know

SECTION C) ALL PARTICIPANTS CHILDREN QUESTIONS (ONE PER CHILD)

Q01. Is your child currently enrolled in in-person learning, where s/he is physically on PKY’s campus in-person? (Yes/No) *Note: your child may have been asked to isolate at home is s/he is a potential contact. Please indicate here the choice you indicated to PKY and where your child is registered as a student.*

Q02. Is your child playing sports this fall?
Yes, No, Haven’t decided

If yes: Which sport? _______________

Past COVID-19 or viral syndrome health event [repeat for each event]

1. Has your child had COVID-19 or a collection of COVID-19 like symptoms since the last time a questionnaire was filled out/ in the last 4 months?

YES, NO, NOT SURE

1A. IF YES, was a COVID-19 swab test (PCR) done? (yes/no)

- if yes date (mm/dd/yy);
- result (positive, negative, indeterminate, unknown)

1B. IF YES was a COVID-19 antibody test done? (yes/no);

- if yes
  - date (mm/dd/yy);
  - result (positive, negative, indeterminate, unknown)
  - type (IgG, total antibody, unknown)

1C. IF YES, was an additional type of COVID-19 testing done other than a swab (PCR) or Antibody? (yes/no);

- if yes
  - date (mm/dd/yy);
  - result (positive, negative, indeterminate, unknown)
  - type (free text)

1D IF YES, was an alternative explanation identified other than COVID-19? (yes/no)

- if yes radio buttons (strep throat, viral influenza, urinary infection, unknown, other);
  - if other, ____ (free text)

1E. IF YES, was care sought from a provider ___________ (yes/no)

- If yes, When____________ (mm/dd/yy)
- If yes, what type of provider was sought (clinic / emergency room without hospitalization / hospitalization / other).
  - Other _____________ (free text)
  - If your child was hospitalized, s/he back at home after discharge? Yes _____ No ___

1F. IF YES Was an inflammatory syndrome diagnosis made (e.g. Kawasaki's Disease, MIS-C)? Y/N

1G. IF YES Was a diagnosis of Toxic Shock made?Y/N

1H. IF YES Do you feel like your child's health is back to normal? Yes /No /Not sure

- If No /Not sure, provide details__________________________ (free text)

1J. If YES: For this 'COVID-19' or 'Viral Syndrome' illness please state what your child's symptoms were:

Symptom	Yes/ No	(IF SYMPTOM YES) Start date	End date (or categorical skip to next variable "still present")
1K.1 Fever* (Branching logic below)			
1K.2 Cough			
1K.3 Fatigue			
1K.4 Shortness of breath			
1K.5 Produces mucus when coughs			
1K.6 Nasal congestion			
1K.7 Sore throat			

1K.8 Headache			
1K.9 Joint or muscle aches			
1K.10 Nausea			
1K.11 Vomiting			
1K.12 Diarrhea (3 or more loose stool in 24 hours)			
1K.13 Abdominal Pain			
1K.14 Loss of sense of taste			
1K.15 Loss of smell			
1K.16 Rash			
1K.17 'COVID toes'			
1K.18 Other (specify)			

*If 'yes' to fever	Answer
Was it measured (yes/no)	
If measured, what method (oral/forehead/ear/armpit)	
Highest measured value of the fever (F)	
Date of fever ended	

1L. IF YES Has your child been seen by his/her physician since resolution of the initial illness?
Yes ______ No ______ Not sure _______

1L.1 If yes, physician name and dates of visits:

____

____

Current health status

2. Independent of past illness, does your child currently have any of the following symptoms? (check for "yes")

Symptom	Present Yes/ No? (for this illness period)	(IF SYMPTOM YES) Start date	End date (or still present)
2A. Fever*			
2B. Cough			
2C. Fatigue			
2D. Shortness of breath			
2E Produces mucus when coughs			
2F Nasal congestion			
2G Sore throat			
2H Headache			
2I Joint or muscle aches			
2J Nausea			
2K Vomiting			
2L Diarrhea (3 or more loose stool in 24 hours)			

2M Abdominal Pain			
2N Loss of sense of taste			
2O Loss of smell			
2P Rash			
2Q. “COVID toes’			
2R. Other (specify)			

*If ‘yes’ to fever	Answer
Was it measured (yes/no)	
If measured, what method (oral/forehead/ear/armpit)	
Measurement value of the fever (F)	
Date of most recent fever	
Date that fever began for this illness period	

Flu

Has your child received a flu vaccine yet for the 2020-2021 flu season?

- Yes
- No
- Not sure

If no/not sure, do you plan on your child getting a flu vaccine (either injection or mist) this season?

- Yes
- No
- Not sure

Household information

1, How many persons live in the child’s household(s) in the period since the last questionnaire (in cases where the child lives between more than one household, please combine the number in both households) _______ (#)

1.A Between how many households does the child live?

2. Since the last questionnaire, has any member of the child’s collective household had COVID-19 with or without lab confirmed test, or had a COVID-19-like viral syndrome of unknown cause?

IF YES questions 2A-2K

2A. If yes, fill in the following information for each event:

2B. Name (First/ Last)

2C. Age (yr)

2C. Sex (F/M)

2D. Date illness started (date)

2E. Is illness resolved (y/n)

2F. If yes, date illness resolved

2G. Was a COVID-19 swab test (PCR) done? (yes/no);

if yes date (mm/dd/yy);

result (pos, neg, indeterminate, unknown)

2H. Was a COVID-19 antibody test done? (yes/no);

if yes date (mm/dd/yy);

result (pos, neg, indeterminate, unknown);
type (IgG, total antibody, unknown)

2I. Was an additional type of COVID-19 testing done other than a swab (PCR) or antibody? (yes/no);

if yes date (mm/dd/yy);
result (pos, neg, indeterminate, unknown);
type (free text)

2J. Was an alternative explanation identified than COVID-19? (yes/no)

If yes radio buttons(strep throat, viral influenza, urinary infection, unknown, other);
if other, ____ (free text)

2K. General comment on the event (free text)

3. Has anyone in the household died from COVID-19? If yes, how many?
4. Has anyone in the family died from COVID-19? If yes, how many? For each deceased family member, list relationship to student.

CHILD STRESS and COPING MECHANISMS

If child is less than 8:
In consultation with your child, please have him/her answer the following questions:

In the past three weeks have you had the following feelings or thoughts? And if so, how often? (Never, A Little, Sometimes, A Lot, or Always; Likert 1-5)

1. I feel hopeless and sad (about the virus)
2. I have trouble eating or sleeping
3. I find myself crying a lot
4. I feel worried or nervous (about the virus)
5. It is hard to stop my thoughts (about the virus)
6. I cannot stop worrying (about the virus)
7. I am very scared of getting dirty
8. I have to wash my hands, over and over to feel better
9. I have a stomachache/headache
I0. It's hard for me to think a long time

Please ask and share your child's thoughts on the following questions:

(Free text)

1. What do you feel when you think of the virus?
2. How can you help yourself feel better?
3. How would you help a friend if they were feeling scared about the virus?
4. How is school helping you with the virus?
5. What else could help you with your feelings about the virus?
6. Would you like to read about ways to feel better about the virus?

If child is 8-13:
Please allow your child to read and answer the following questions his/herself :

In the past three weeks have you had the following feelings or thoughts? And if so, how often? (Never, A Little, Sometimes, A Lot, or Always; Likert 1-5)

1. I feel hopeless and sad (about the virus)
2. I have trouble eating or sleeping
3. I find myself crying a lot
4. I feel worried or nervous (about the virus)
5. It is hard to stop my thoughts (about the virus)
6. I cannot stop worrying (about the virus)
7. I am very scared of getting dirty
8. I have to wash my hands, over and over to feel better
9. I have a stomachache/headache
I0. It's hard for me to think a long time

Please share your thoughts on the following questions:
(Free text)

1. What do you feel when you think of the virus?
2. How can you help yourself feel better?
3. How would you help a friend if they were feeling scared about the virus?
4. How is school helping you with the virus?
5. What else could help you with your feelings about the virus?
6. Would you like to read about ways to feel better about the virus?

If child is over 13:
Please allow your child to read and answer the following questions his/herself :

In the past three weeks have you had the following feelings or thoughts? (Never, Occasionally, Half the Time, Often, Always; 1-5))

1. Sadness, feeling down, low mood, feeling fatigued
2. Feelings of hopelessness, worthlessness, emptiness, or not being a good person
3. Decreased pleasure from things that used to be fun, feeling that life is not much fun
4. Feeling worried, nervous, panicky, tense, stressed out
5. Not being able to stop worrying or controlling your worry
6. Being easily annoyed or irritable, feelings of dread like something awful might happen
7. Constant thoughts about avoiding germs
8. Fixation with washing your hands throughout the day
9. Sudden moments of fear or terror because you couldn't get rid of the germs
10. Felt a racing heart, shaky, sweaty, or had trouble breathing

Please share your thoughts about the following questions:
(Free text)

1. If you feel stressed or scared about the coronavirus, what seems to help you feel better?
2. How do you help yourself feel calm when you hear about how the virus is spreading?
3. How do you help your friends that are feeling scared about the coronavirus?
4. What parts of school are helping you feel supported about your feelings about the coronavirus?

5. How does continuing to see your peers and teachers help you during this pandemic?
6. What types of handouts or readings would you be interested in about the coronavirus?
